# What factors do patients consider when choosing a hand surgeon?

**DOI:** 10.1080/23320885.2023.2222802

**Published:** 2023-06-16

**Authors:** Monika Debkowska, Brian Lynch, Jeremy Ruskin, David Komatsu, Samuel Caradonna, Edward Wang, Samantha Muhlrad

**Affiliations:** Department of Orthopaedic Surgery, Stony Brook University Hospital, Stony Brook, NY, USA

**Keywords:** Hand, surgery, patients, office, referrals, demographics

## Abstract

This study evaluated what patients consider in choosing a surgeon. A survey was given to 303 patients. Most found their hand surgeon through a medical or family/friend referral (*p* < .05). Surgeon credentials and accepted insurance were statistically more likely to be rated first (*p*<.001). We recommend educating referring physicians about our practices.

## Introduction

In the United States, there are over 3800 plastic and orthopedic hand surgeons registered with the American Society for Surgery of the Hand, the largest hand surgical society in the country [1]. The large number of specialists can present a dilemma for patients, as there is little information to help them decide whom to see for surgical and nonsurgical issues. The factors that patient’s take into consideration when choosing a hand surgeon are important to understand so that surgeons can best address their need. In 2016, there were over 30 million outpatient physician visits to hand specialists [2]. Not all patients find or are referred to the correct specialist on their first attempt and may find themselves frustrated wasting time and money just to be referred to another specialist more suitable for them [3]. Surgeons would benefit from learning how to optimize their practice to reach their appropriate patient population within their specialty for maximum benefit and minimum resource waste, both for the patients and for the physicians.

During training, hand surgeons are taught the intricacies of their craft, learning how to perform complex surgery down to the most minute detail. There is little to no education on practice management during the training period. Bozic et al. found that only 46% of patients were able to find useful comparative information with regards to patient outcomes between surgeons, so the ability to consider patient skill in choosing a surgeon is limited [4]. Surgeons may not realize some of the important factors that patients are concerned about. Bozic et al. also showed that patients considering total joint arthroplasty valued physician skill nearly equally to bedside manner when choosing a surgeon, in comparison to multiple other factors [4].

In 2014, Abghari et al. published their results on patient perceptions and preferences when choosing a surgeon [5]. Their study focused on specific characteristics of the surgeon, such as their demographics, professional standing and physical attributes. The results demonstrated that most patients did not value age, ethnicity, religion or gender of their surgical specialist. They did find that patients who care about race and religion tended to favor surgeons within their same group. Besides surgeon specific factors, finding a specialist may be also dependent on other variables, like their insurance company. Manning et al. and Fady et al. both found that patients relied on in network health insurance plans when seeking a spine specialist [6,7].

Despite the limited research looking into the field of hand surgery, there have been no studies to date looking at the factors that patients consider when choosing a hand surgeon. We therefore propose a survey study to evaluate which factors patients consider important when choosing a hand surgeon and if these factors differ based on the participants’ demographics. Our hypothesis is that patients of differing age groups and gender will value different factors that they believe are most important when choosing their hand surgeon.

## Methods

This was a cross-sectional survey study conducted at two medical institutions on Long Island, New York: Stony Brook University Medical Center and Orthopaedic Associates of Long Island. A questionnaire (see Appendix) was developed including four closed-ended questions and one-open ended question. It was developed based on discussions between hand surgeons, staff and patients. The survey included demographic questions, such as age and gender. The remaining questions evaluated how the participants chose their hand surgeon and what factors influenced this decision. The survey was handed out by clerical staff to all new patients being evaluated by a hand surgeon at the three medical institutions between January 2021 and February 2021. All seven orthopedic and plastic hand surgeons participated in the research study. All seven surgeons were part of the same practice and all had patient surveys completed. No one had individual advertising. Following completion of the survey, participants placed their survey into a Manila envelope. For patients who were under 18 years of age, the surveys were filled out by their parents or legal guardians. Patients who were following up from the Emergency Department were excluded, as the hand surgeon they were seeing was predetermined by the hand surgery call schedule. The questionnaire was completely anonymous and did not include identifying information. Because of this we did not have access to their socioeconomic status, race, education background or healthcare insurance. Willingness to participate in the study was voluntary. Participants were able to opt out of the survey at any time and had the option to skip any questions. Answers were compared based on gender and age demographics. We further analyzed which factors affected participants the most when choosing their hand surgeon. Descriptive data analysis, including percentages and frequencies, was conducted. Differences between groups were evaluated using Chi-squared tests. All analyses were performed using Prism Ver. 9.31.0 (GraphPad Software, LLC.) The alpha value or threshold for statistical significance was set to 0.05. This study was approved and monitored by the Stony Brook University Hospital Institutional Review Board. Consent forms were not required.

## Results

At the completion of the two-month collection period, there were 303 survey respondents out of 593 new patients seen by our seven hand surgery physicians, making the response rate 51%. There were 129 males and 174 females that completed the survey (Table 1). Of the 303 respondents, 68 were above the age of 66 years old, 164 were between the ages of 41 and 65 years old and 58 were between the ages of 19 and 40 years old (Table 1). There were 13 participants whose parents completed the survey (Table 1). Analysis of the demographic data showed no significant differences between the number of male and female respondents within each age category.

**Table 1.** Survey participants demographics.

Gender	Number of participants	Percentage of participants (%)
Male	129	42.5%
Female	174	57.4%
Age		
0–18 years	13	4.3%
19–40 years	58	19.1%
41–65 years	164	54.1%
>65 years	68	22.4%

**Figure 1.**
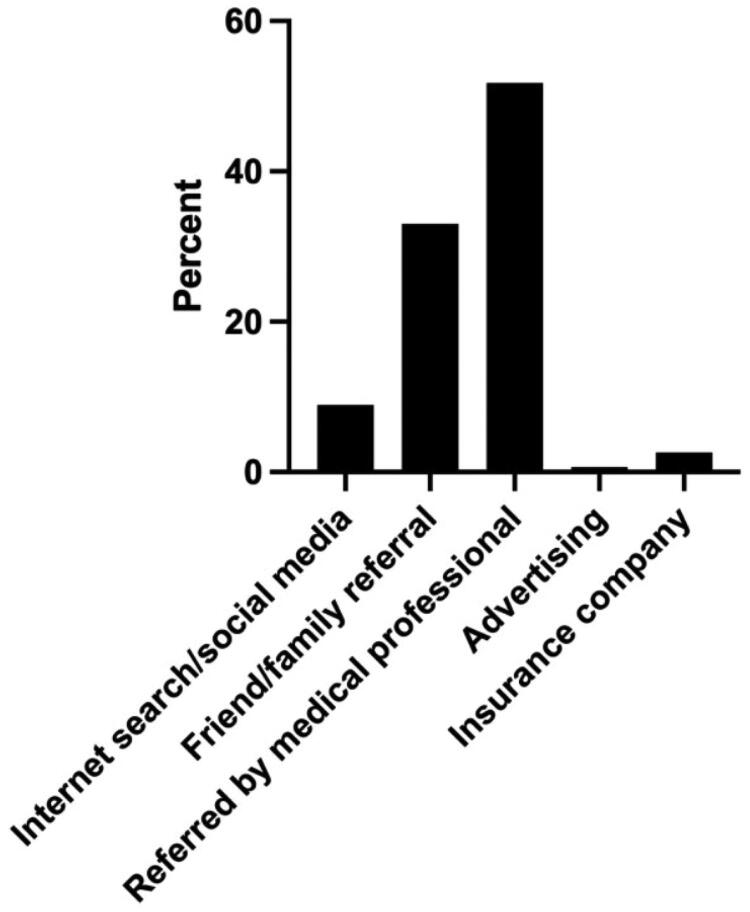
How survey participants found their hand surgeon’s offices.

Next, we asked the survey respondents how they found their hand surgeon’s office (Table 2, Figure 1). Most patients (87%) found their hand specialist through a medical professional or family/friend referral, as compared to advertisement, social media and insurance company (*p* < .05). The majority of respondents was referred by a medical professional (52.5%). The second most common reason was referral by a friend or family member (34.9%). Only 9.3% of participants used social media or an internet search to find their hand surgery specialist. The least common ways that respondents found their hand surgeon was through using their insurance companies (2.7%) or through advertising (0.7%).

**Table 2.** How survey participants found their hand surgeon’s offices.

	Total number of participants	Percentage of participants (%)
Internet search/social media	28	9.3%
Friend/family referral	105	34.9%
Referred by medical professional	158	52.5%
Advertising	2	0.7%
Insurance company	8	2.7%

**Figure 2.**
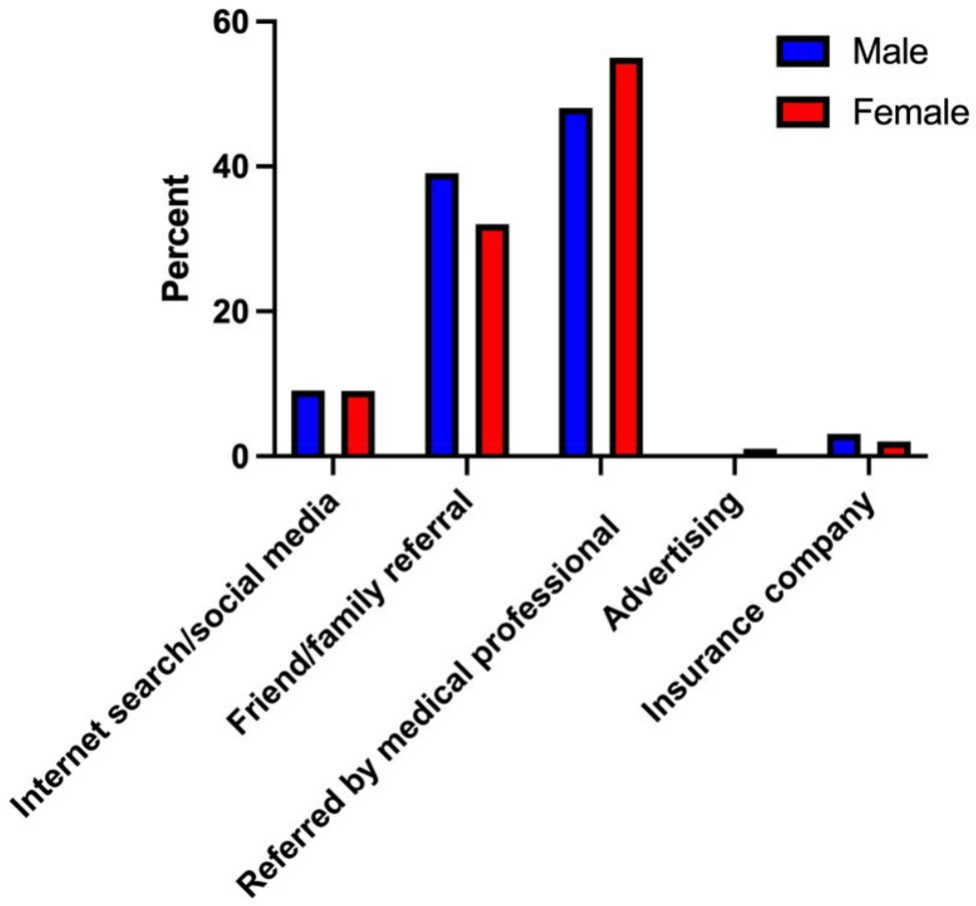
How survey participants found their hand surgeon’s offices based on gender.

When analyzing how participants found their hand surgeon’s office based on gender there were no significant differences (Table 3, Figure 2). 55.5% of females and 48.4% of males were referred to their hand surgeon by a medical professional. 31.8% of females and 39.1% of males were referred to their hand surgeon by a family or friend. 9% of females and 9% of males used social media or an internet search to find their hand surgery specialist, while 2.3% of females and 3.1% of males used their insurance companies. Only 1.2% of females and no males used advertising to find their hand surgery specialist.

**Table 3.** How survey participants found their hand surgeon’s offices based on gender.

	Male	Female	Male %	Female %
Internet search/social media	12	16	9.4	9.3
Friend/family referral	50	55	39.1	31.8
Referred by medical professional	62	96	48.4	55.5
Advertising	0	2	0	1.2
Insurance company	4	4	3.1	2.3

**Figure 3.**
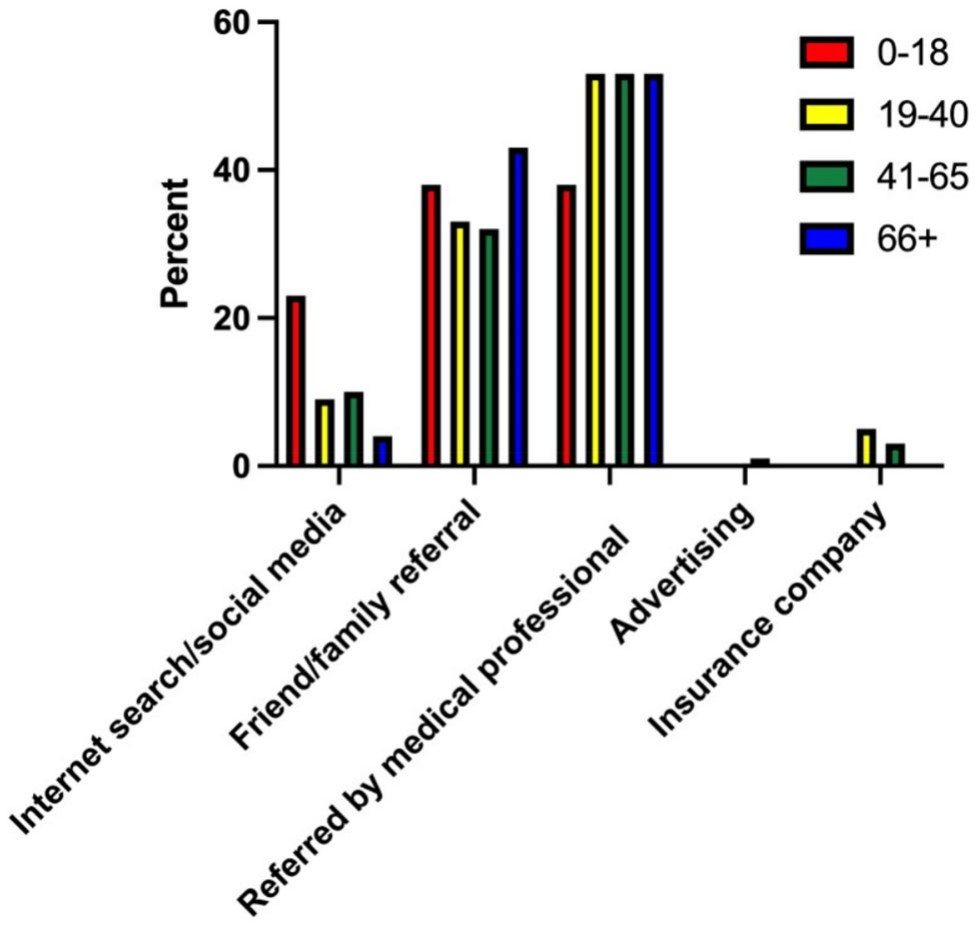
How survey participants found their hand surgeon’s offices based on age.

In terms of different age categories, there were no significant differences in how participants found their hand surgeon’s office (Table 4, Figure 3). Participants greater than 65 years of age used mainly referrals from medical professionals (52.9%) or referrals from family and friends (42.7%) and none used advertisement or went through their insurance companies. Only 4.4% of participants greater than 65 years of age used social media or an internet search to find their hand surgery specialist. Respondents between 41 and 65 years of age also mainly used referrals from medical professionals (53.1%) or referrals from family and friends (32.1%). While only 10.5% of respondents between 41 and 65 years of age used social media or an internet search, only 3.1% used their insurance companies and only 1.2% used advertisement. This was similar to patients between 19 and 40 years of age, in which 53.4% used referrals from medical professionals and 32.8% used referrals from family and friends. 8.6% of respondents between 41 and 65 years of age used social media or an internet search, while 5.2% used their insurance companies to find their hand surgeon. No participants between 19 and 40 years of age used advertisement to find their hand surgery specialist. Lastly, the parents of patients under the age of 18 years mainly used referrals from medical professionals (38.5%) and referrals from family and friends (38.5%). 23.1% of parents used social media or an internet search, while no parents used their insurance companies or advertisement.

**Table 4.** How survey participants found their hand surgeon’s offices based on age.

	Age (years)			
	0–18 (%)	19–40 (%)	41–65 (%)	>66 (%)
Internet search/social media	23.1	8.6	10.5	4.4
Friend/family referral	38.5	32.8	32.1	42.7
Referred by medical professional	38.5	535	53.1	52.9
Advertising	0	0	1.2	0
Insurance company	0	5.1	3.1	0

**Table 5.** Top three reasons participants ranked when choosing their hand surgeon (%).

Rank	Surgeon credentials/ schooling	Social media	Available appointments	Office location	Online ratings/ reviews	Surgeon gender	Surgeon nationality	Surgeon years of experience	Accepted insurance plans
1st	50.8	3.2	19	16.7	16.2	3.4	0	29.6	47
2nd	13.2	0	14.5	12.1	14.7	1.1	1.2	29.6	16.3
3rd	10.1	2.1	12.3	16.1	14.7	0	0	19	19.8

Next, we analyzed nine different factors that patients used when looking for their hand surgery specialist (Appendix). Not all factors were ranked by every respondent. The factors more commonly ranked were accepted insurance plans ranked by 202 out of 303 respondents, surgeon schooling ranked by 189 respondents, available appointments ranked by 170 respondents, years of experience also ranked by 179 respondents and office location ranked by 174 respondents. Online reviews and ratings were ranked by 136 respondents, while social media was ranked by 95 respondents. The factors least commonly ranked were surgeon gender by 88 respondents and surgeon nationality by 85 respondents.

**Figure 4.**
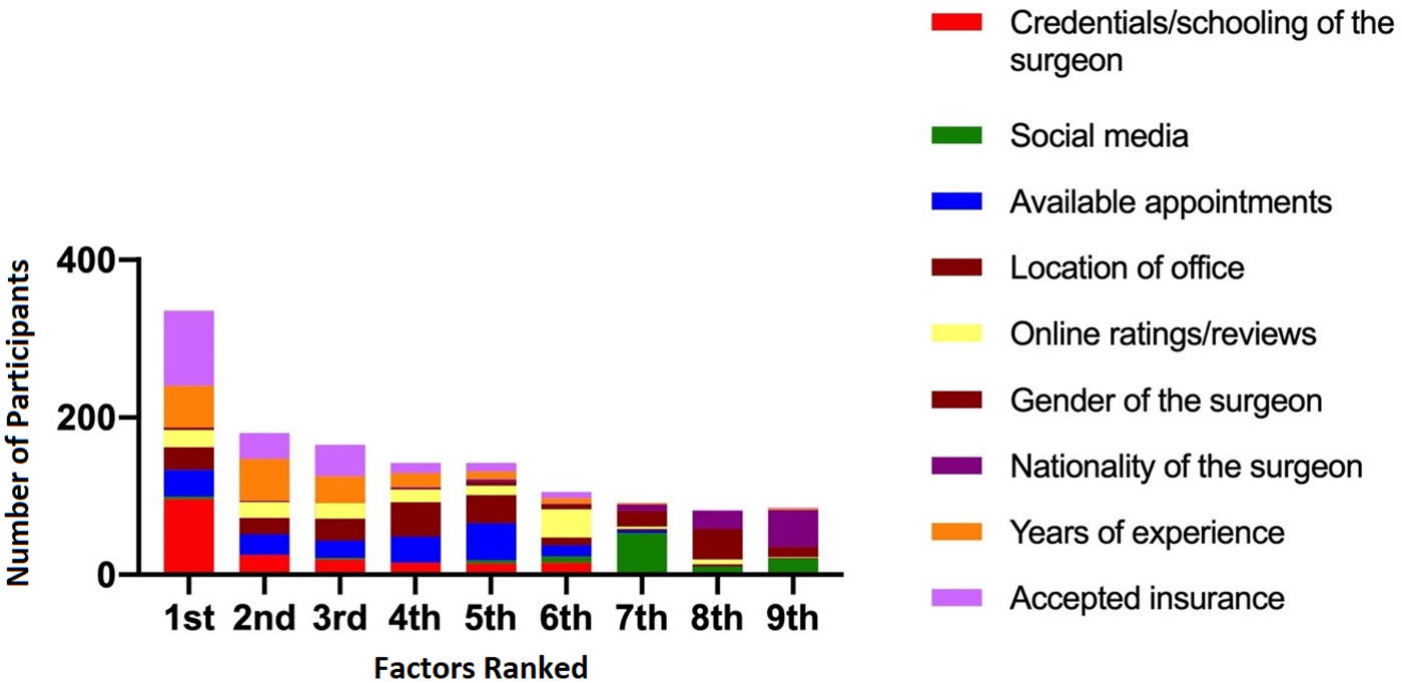
Factors ranked by survey participants from most to least important when choosing a hand surgeon.

When ranking these various factors, surgeon credentials and accepted insurance plans were statistically more likely to be rated first as compared to available appointment times, office location, social media information, online reviews/ratings, surgeon gender, surgeon nationality or surgeon years of experience (*p*<.001). Overall, the factors that were ranked higher more frequently were surgeon credentials and schooling, available appointment times, office location, online ratings and reviews, surgeon years of experience and accepted insurance plans (Figure 4). While the factors that were ranked lower more frequently were social media, surgeon gender and surgeon nationality (Figure 4). Lastly, there were no significant differences between male and female patients and between different age groups when ranking these various factors.

**Figure 5.**
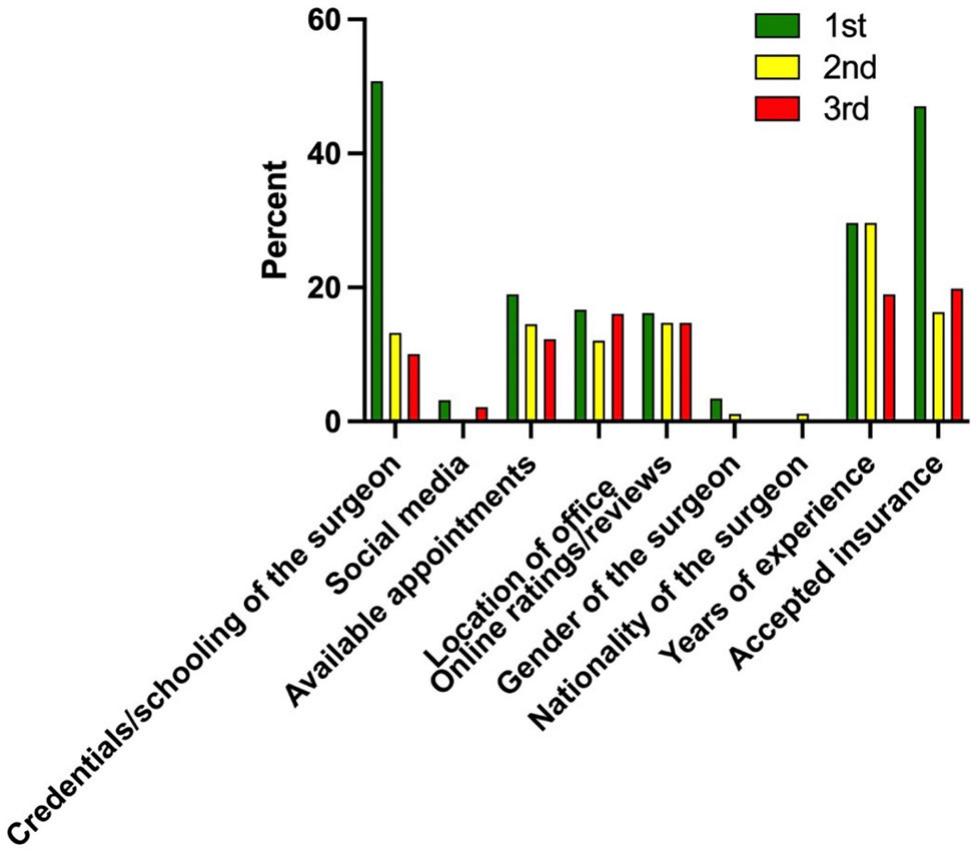
Top three reasons participants ranked when choosing their hand surgeon.

Figure 5 shows the top three most important factors that respondents ranked when choosing their hand surgery specialist. 50.8% of participants ranked surgeon credentials or schooling as the most important reason for choosing their hand surgeon, 47% ranked accepted insurance plans as the most important factor (Table 5). Also ranked first but not statistically significant as the most important factor was surgeon years of experience, ranked by 30% of participants. The most common factor ranked second was surgeon years of experience (29.6%) followed by accepted insurance plans (16.3%). The most common factor ranked third was accepted insurance plans (19.8%) followed by surgeon years of experience (19%). The factor ranked least for most important reason when choosing a hand surgeon was surgeon nationality, which was not ranked by any participant. Only 3.4% of participants ranked surgeon gender as the most important factor when choosing a hand surgeon.

When asked if there were any other factors that influenced the respondent’s decision when choosing their hand surgeon, most patients wanted a doctor that was collegial, patient, had good manners, and overall was a good, friendly doctor. A respondent wrote that is it important for their hand surgeon to be ‘nice and seem like they generally care’. A few respondents added that they preferred a competent doctor that was experienced in surgery with a good reputation. Some participants wrote that social media, surgeon gender and surgeon nationality ‘do not matter’.

## Discussion

There has been little research into the area of patient decision making when choosing which surgical specialist to see in the outpatient setting. This is the first survey study analyzing the most important reasons for patients when choosing their hand surgeon. Furthermore, we focused on demographics including age and gender to determine if these influenced the respondents’ answers. Contrary to our hypothesis, patients regardless of their age or gender share similar reasons for choosing hand surgeons at our clinical offices in New York. We also found that surgeon gender and surgeon nationality were ranked lowest out of nine factors when choosing the most important factors for finding a hand surgeon. Some participants even added to the comments section that gender does not matter when choosing a hand surgeon.

We found that at our academic institution most patients found their hand surgeon through medical or family and friend referrals. This traditional route of finding a physician, especially a specialist, has not changed in the past two decades. In 2007, in a national survey presented from The Center for Studying Health System Change reported that half of adults relied on word of mouth from friends and family to find a new primary care physician, while 68.5% of adults used a referral from their primary care physician to find a specialist [8]. Similarly, a web-based survey study conducted in 2008 by Freed et al. found that most respondents (84%) reported that recommendations from friends and family were very important factors when choosing a physician for their child [9]. As many of our survey respondents commented that they wanted a good, friendly doctor and as a large number of respondents found their hand surgeon through family and friend referral, it is important to establish a good rapport with our patients. Additionally, we recommend educating referring physicians about the practices of our specialty as this was the most important reason for how our respondents found their hand surgeon’s clinical offices. This may decrease patient frustration as well as time and money wasted if they are referred to the wrong specialist for their hand concerns.

In terms of factors that influenced patients to choose their hand surgeon, we found that surgeon credentials and accepted insurance plans were the two most common factors out of the nine listed in our survey. Similar to our findings, in two studies conducted by Manning et al. looking at patients choosing foot and ankle specialists as well as sports medicine surgeons showed that out of 19 factors listed the patients preferred board certification, availability of onsite imaging and in-network insurance coverage when choosing their specialist [10,11]. Therefore, it may be beneficial to expand health insurance coverage plans to help obtain patients.

Many practitioners spend valuable time and money on advertising their practices. Similar to our findings, Manning et al. also found that out of 19 factors listed the least important factors included the effect of internet, radio or TV advertisements [10,11]. As very few patients in our study used advertisements to find their hand specialist, we caution similar practices in academic settings against overspending their resources on print and radio advertisements. Furthermore, with the new social media rise over the last two decades many physicians spend time creating websites to publicize or market their practices. In our study, we found very few respondents that used social media to find their hand surgeon, even in all age categories. Therefore, hand specialists in academic institutions in rural or suburban settings may not need to spend time or their resources creating social media pages for their practices. This is also significant as social media websites that do not have their content protected from patient information, including photos or images, may result in HIPAA violations.

## Strengths

One of the strengths of our survey study was the high response rate. A typical response rate for a survey research study is approximately 60% [12]. Our response rate was 51%. Therefore, our results are generalizable and representative of the population of interest. Another strength of the study was that it was completed at multiple different offices, allowing us to obtain responses from various patient populations, including suburban and urban patient populations. We also included plastic and orthopedic hand surgeons in the study.

## Limitations

One of our limitations was incomplete participant response to questions number four, which included ranking factors of most to least importance when choosing their hand surgeon. Although not every participant ranked all factors listed, we were still able to gather which factors were most important in influencing patient decision when choosing their hand specialist. There are also inherent limitations to a survey-type questionnaire, such as social desirability bias in which participants answers questions based on how their answered would be viewed by others instead of answering truthfully. However, all surveys were anonymous and collected at the clerical desk in a Manila envelope, preventing any health care professional from seeing the participant’s responses. Since this study was anonymous we were not aware of socioeconomic status, race, education background or healthcare insurance carrier which could play a role in their responses. Lastly, our institution has a poor social media presence which may have influenced participant answers when not ranking social media as an important reason for choosing their hand surgeon specialist.

## Future direction

We plan on expanding the survey to the entire Department. We also plan on surveying the surgical faculty to determine if their perspectives on the most important factors when choosing a specialist differ from their patients. Lastly, we think it would be interesting to include private practice centers as well as centers in rural areas to determine if there are differing factors that influence patients when they are choosing their hand surgeon.

## Conclusions

Overall, this study helps clarify how patients choose their hand surgeon. Contrary to our hypothesis, patients regardless of their age or gender share similar reasons for choosing hand surgeons. Although in the age dominated by the internet and online resources, at our academic institution in New York we found that most patients continue to rely on traditional approaches to find their specialist through medical or family/friend referrals.

## Consent form

Consent was waived as it was a voluntary anonymous survey. Statement of Human and Animal Rights – No rights were violated

## Appendix

Dear participant,

The following survey is for quality control and patient experience improvement. It will be anonymous and will not affect the quality of care that you receive. It is completely voluntary and any responses are appreciated. Responses may be used for research purposes in order to improve the future care that patients receive.

Thank you.

Approved by the IRB in December 2020.

1. How did you find the doctor’s office when looking for a hand specialist?

- Internet search/social media
- Friend/family referral
- Referred by medical professional
- Advertising
- Insurance company

_____________________________________________

2. Gender

- Male
- Female

_____________________________________________

3. What is your age?

- 0–18
- 19–40
- 41–65
- 66+

_____________________________________________

4. Please rank the following in order of importance when choosing a hand specialist to see with 1 being most important:
 __ Credentials/schooling of the surgeon
 __ Social media
 __ Available appointments
 __ Location of office
 __ Online ratings/reviews
 __ Gender of the surgeon 
 __ Nationality of the surgeon 
 __ Years of experience 
 __ Accepted insurance

_____________________________________________

5. Are there any other factors that you would have ranked either 1 or 2 if they were included as choices in question 4? Please include here.
